# Nitric oxide-mediated the therapeutic properties of induced pluripotent stem cell for paraquat-induced acute lung injury

**DOI:** 10.3389/fimmu.2023.1136290

**Published:** 2023-05-18

**Authors:** Anfeng Cui, Shirui Li, Yijun Li, Dawei Yang, Jiongwei Huang, Xuemeng Wang, Nana Song, Fuchen Chen, Sifeng Chen, Meng Xiang

**Affiliations:** ^1^ Department of Physiology and Pathophysiology, School of Basic Medical Sciences, Fudan University, Shanghai, China; ^2^ Department of Pathology, The Second Clinical Medical College, Shanxi Medical University, Taiyuan, China; ^3^ Department of Pulmonary and Critical Care Medicine, Zhongshan Hospital Fudan University, Shanghai, China; ^4^ Health Management Center Department, Zhongshan Hospital, Fudan University, Shanghai, China; ^5^ Fudan Zhang Jiang Institute, Shanghai, China

**Keywords:** induced pluripotent stem cell, nitric oxide, acute lung injury, L-arginine, L-NAME

## Abstract

The mortality rate associated with acute lung injury (ALI) and its severe form, acute respiratory distress syndrome, is high. Induced pluripotent stem cell (iPSC) therapy is a potential treatment method for ALI, but its therapeutic efficacy is limited in injured lungs. Nitric oxide (NO) has various physiological actions. The current study investigated the effect of iPSCs pretreated with NO donors in paraquat (PQ)-induced ALI mouse model. Male C57BL/6 mice were intraperitoneally injected with PQ, followed by infusion of phosphate-buffered saline, iPSCs, L-arginine pretreated iPSCs, or Nitro-L-arginine methylester (L-NAME) pretreated iPSCs through the tail veins. Histopathological changes, pulmonary microvascular permeability, and inflammatory cytokine levels were analyzed after 3 or 28 d. The effects on iPSC proliferation, migration, and adhesion were evaluated *in vitro*. More L-arginine-pretreated iPSCs were selectively trafficked into the injured pulmonary tissue of mice with LPS-induced ALI, drastically diminishing the histopathologic changes and inflammatory cytokine levels (IL-1β and IL-6). There was also markedly improved pulmonary microvascular permeability and pulmonary function. The NO inhibitor abolished the protective effects of iPSCs. In addition, the ability of L-arginine to promote the proliferation and migration of iPSCs was decreased by L-NAME pretreatment, suggesting that NO might mediate the therapeutic benefits of iPSC. The improvement of the iPSC physiological changes by the endogenous gaseous molecule NO reduces lung injury severity. L-Arginine represents a pharmacologically important strategy for enhancing the therapeutic potential of iPSCs.

## 1 Introduction

Acute lung injury (ALI) is characterized by noncardiogenic pulmonary edema resulting from the disruption of endothelial cells and the alveolar-capillary barrier, which occurs due to excessive pulmonary and systemic inflammation (1). The adult lung has a remarkably low level of cellular turnover as it is a highly quiescent tissue. The intrinsic complexity of the lung includes at least 40 different cell phenotypes from the trachea to the alveolar space(2). Despite substantial advances in supportive measures, there are no specific pharmacological treatments to halt or reverse the development or progression of ALI (3). The overall mortality rate of ALI is 38% in the United States of America (4). Therefore, exploring multi-targeted anti-ALI/ARDS therapies is critical.

Induced pluripotent stem cells (iPSCs) are stem cells reprogrammed from somatic cells with unlimited proliferative potential; and hold great therapeutic potential for most major diseases, including acute lung injury (ALI) (5) (6). In 2019, we used 53 conditions and animal disease models to demonstrate that disseminated iPSCs are influenced by the microenvironment *in vivo* and completely avoid teratoma formation (7). Our recent work and other studies reported that iPSCs transplantation reduces neutrophil chemotaxis, improves associated pathological changes, and alleviates lung injury (5, 8). However, the duration of the therapeutic effect is limited, and the number of injected iPSCs decreased by 80%, 28 d after administration (7). The pathophysiological microenvironment (such as inflammatory, hypoxic, and/or ischemic microenvironments) diminishes stem cell survival, thus inhibiting their therapeutic efficacy (9). Therefore, strategies to develop *in vitro* drugs that enhance stem cell function and increase therapeutic efficacy is required.

Nitric oxide (NO), a diatomic free radical, is one of the most important signaling molecules in mammalian physiology that modulates physiological actions within the nervous, cardiovascular, and immune systems. NO-based therapeutics are widely studied as pharmacological intervention treatments of cancers, cardiovascular, and neural diseases (10). Several studies have utilized inhaled NO to manage abnormal pulmonary vascular tones and improve oxygenation in patients with ALI or ARDS; NO did not reduce mortality and even caused organ damage (11, 12). The study of NO *in vivo* is important because it is a multifunctional signaling molecule that plays important roles in regulating the growth, survival, apoptosis, proliferation, and differentiation of many cells (13). In addition, previous studies found that NO is involved in stem cell epigenetic regulation, differentiation, and immune suppression (14, 15). However, the NO-mediated regulation of the therapeutic properties of transplanted iPSCs have not to be elucidated.

Therefore, in this study, we explored the influence of NO precursor (L-arginine) on iPSC behavior and anti-inflammatory and antifibrotic effects on lung injury *in vivo*.

## 2 Materials and methods

### 2.1 Mouse strain

50 Male C57BL/6 mice (aged 8-10 weeks) were purchased from the SLAC Laboratory Animal Co., Ltd. (Shanghai, China) and housed in an animal facility at Fudan University. They were fed standard rodent chow and given water ad libitum. All animal protocols adhered to the criteria of the National Research Council’s Guide for the Care and Use of Laboratory Animals and were approved by the animal care committee of Fudan University Shanghai Medical College.

### 2.2 Mouse iPSC culture

C57BL/6 mouse iPSCs were induced from mouse embryonic fibroblasts (MEFs), derived from 13.5-day-old embryos, by lentivirus vectors carrying Yamanaka factors, as previously described (5). iPSCs were routinely cultured and expanded under feeder-free conditions with the use of multiple small-molecular inhibitors (CHIR99021 and PD0325901). Briefly, a standard iPSCs medium consisted of Dulbecco’s Modified Eagle’s Medium (DMEM) supplemented with 0.1 mM non-essential amino acids, 1 mM sodium pyruvate, 0.05 mM b-mercaptoethanol, 50 U/ml penicillin, 50 mg/ml streptomycin, 0.1 mg/ml leukemia inhibitory factor, 0.5 mg/ml CHIR99021, 0.5 mg/ml PD0325901, and 10% fetal bovine serum. iPSCs was suspended in the standard iPSC culture medium and seeded into 6-well plates pretreated with 0.2% gelatin. Cells were stimulated with L-arginine (L-Arg, 100 µM, Sigma, USA) or N-nitro-L-arginine methyl ester (L-NAME, 100 µM, Sigma, USA) for 2 hours.

### 2.3 iPSCs quantification *in vivo*

The external *Tet-on* gene can be used as a marker for iPSC identification and quantification. To observe the homing and survival of injected syngeneic iPSCs in the injured lungs, the lung tissue was harvested on days 3 and 28 post—iPSC administration, and the *Tet-on* level was measured. 18S rDNA expression was used as an internal control. For each time point, n = 5. The Tet-On-specific PCR primers: sense, 5’-AGCACAACTACGCCGCACCC-3’; antisense, 5’-ATGCACCAGAGTTTCGAAGC-3’.

### 2.4 Paraquat-induced lung injury

Paraquat (PQ, 1,1’-dimethyl-4,4’-bipyridylium dichloride) mainly accumulates in the pulmonary tissue, and causes ALI/ARDS, which is associated with oxidative stress, inflammatory response, and epigenetic alterations etc. The repair process of lung injury can develop into pulmonary fibrosis (16, 17). Thus, we utilized a paraquat-induced lung injury model to evaluate the effect of iPSCs therapy (7). In brief, PQ (Sigma-Aldrich, St Louis, MO) was injected intraperitoneally (15 mg/kg body weight). Two hours post PQ injection, 5×10^6^ iPSCs/kg were injected via the tail vein, and mice were monitored on days 3 and 28. The five experimental groups were: (1) Sham, (2) PQ + PBS, (3) PQ + iPSC, (4) PQ + iPSC pretreated with100 µM L-Arg for 2h, and (5) PQ + iPSC pretreated with 100 µM L-NAME for 2h.

### 2.5 Pathological examination and Masson’s trichrome staining

On days 3 and 28 post-PQ intraperitoneal injection, the left upper lung tissues were fixed in 4% paraformaldehyde for 24 h and embedded in paraffin fixation. Then, the samples were sectioned into the 5-μm thick sections, and hematoxylin and eosin and Masson’s trichrome staining were performed to evaluate the fibers level. Pulmonary sections were photographed by light microscopic fields (Leica SCN400, Leica Microsystems, Wetzlar, Germany), and the semi-quantification of collagen fibrosis was assessed by ImageJ Software version 1.49a (National Institutes of Health, Bethesda, Maryland, USA).

### 2.6 Fluorescent labeling and observation of iPSCs

To track the cells *in vivo*, iPSCs were labeled with the red fluorescent dye PKH26 and injected via the tail vein. The PKH26-labeled iPSCs were observed in frozen sections using a Leica fluorescent microscope (Leica SCN400, Leica Microsystems, Wetzlar, Germany). 4’,6-diamidino-2-phenylindole (DAPI) as used to counter-stain nuclei.

### 2.7 The myeloperoxidase activity in the lung

Myeloperoxidase (MPO) is the principal enzyme of polymorphonuclear leukocytes (PMN) which represents a direct correlation with PMNs infiltration in lung tissue. The lung myeloperoxidase (MPO) activity was measured with an MPO testing kit (A 044, Jiancheng Bioengineering Institute, Nanjing, the People’s Republic of China). In brief, 0.02 g lung tissue was homogenized in 0.2 mL PBS, then centrifuged at 12,000 rpm at 4°C for 20 min. The supernatant (90 μL) was added to Reagent III (10 μL) and incubated in a water bath at 37°C for 15 min. We added 20 μL of the sample to the test and control tube, incubated in a water bath at 37°C for 30 min. MPO activity was measured immediately with the absorbance at 460 nm. The activity was expressed as units per gram of total protein (U/g).

### 2.8 Measurement of total protein, cell concentration in bronchoalveolar lavage fluid

The FITC-BSA (2 mg/kg body weight; Biosynthesis Biotechnology Co., Ltd.) was injected via the tail vein, at 30 min before sacrificing the animals. The left lung lobe was lavaged five times with 200 ml PBS. The bronchoalveolar lavage fluid (BALF) was centrifuged at 200 g × 15* min*. The concentration of FITC-BSA in the BALF was detected via a fluorescent microplate reader (Synergy H1, BioTek Instruments, Inc., Winooski, VT, USA). The total number of nucleated cells in the BALF was also counted.

### 2.9 Measurement of cytokine expression in injured lung

Messenger RNA of interleukin-1β, interleukin-6, and interleukin-10 (IL-1β, IL-6, and IL-10, respectively) were measured by quantitative reverse transcription PCR (RT-qPCR) on day 3 and 28 after lung injury was induced. According to the manufacturer’s instructions, total RNA from lung tissue was isolated using TRI-reagent (Thermo fisher Scientific™, CA, USA). In brief, RNA (2 μg) was reverse-transcribed using random-sequence primers with a ReverTraAce^®^ qPCR RT Kit (TOYOBO CO., Japan). The amplification reactions proceeded as follows: 35 cycles of 20 s at 95 °C, 45 s at 57 °C, 30 s at 72 °C, after initial denaturation at 95 °C for 5 min with 1X SYBR^®^ Green PCR Master Mix (TOYOBO CO., Japan). The relative expression of target genes was calculated by the 2^-ΔCT^ method. The primers for all genes synthesized by Sunny Biological Technology Co. (Shanghai, China) and sequences were as follows:

IL-1β: forward 5’-TGCCACCTTTTGACAGTGATG-3’;
reverse 5’-AAGGTCCACGGGAAAGACAC-3’;
IL-6: forward 5’ - GCCTTCTTGGGACTGATGCT -3’;
reverse 5’ -TGCCATTGCACAACTCTTTTCT-3’;
IL-10: forward 5’ - GGTTGCCAAGCCTTATCGGA -3’;
reverse 5’ -GGGGAGAAATCGATGACAGC-3’;
18 s: forward 5’ - GAGAAACGGCTACCACATCC -3’;
reverse 5’ - CACCAGACTTGCCCTCCA -3’.

### 2.10 Cell viability, adhesion, and migration

iPSCs were treated with 100 μM L-Arginine or 100 μM L-NAME for 2 h, respectively, then added to a 96-well plate (1×10^4^ cells/well). Untreated iPSCs served as controls. iPSCs viability was evaluated at 0, 24, 48, and 72 h using the Cell Counting Kit-8 (Dojindo Molecular Technologies Inc., Shanghai, China) according to the manufacturer’s instructions. The experiment was independently repeated five times.

To explore the effect of NO on iPSCs adhesion, iPSCs labeled with PKH26 red fluorescent dye (Sigma-Aldrich/Merck Group, St. Louis, MO, USA) was pretreated with 100 μM L-Arginine or 100 μM L-NAME for 2 h, respectively. A suspension of the labeled cells (5 000 cells/well) was cultured in a Matrigel™-coated 96-well plate (BD Biosciences, Franklin Lakes, NJ, USA). After one hour of incubation, cells not attached were removed by gently washing the plate three times with PBS. Adherent cells were photographed with a fluorescence microscope (Leica) and counted. The assays were independently repeated five times.

A cell migration assay was performed as follows: iPSC labeled with the red fluorescent dye PKH26 was pretreated with 100 μM L-Arginine or 100 μM L-NAME for 2 h, respectively, and cells (1×10^4^ cells/well) were plated on 24-well Transwell™ permeable supports with porous filters (Corning Inc., Corning, NY) and incubated for 16 h. The fluorescence-positive migrated cells were photographed with a fluorescence microscope and analyzed via ImageJ software (the National Institutes of Health, Bethesda, MD, USA). The experiment was independently repeated five times.

### 2.11 Statistical analysis

The results are presented as the mean ± SD. The *post-hoc* comparison tests were performed with ANOVA to determine statistical differences among multiple groups, and statistical significance was set at *P* < 0.05. Data are representative of five independent experiments.

## 3 Results

### 3.1 NO is necessary for iPSCs to improve PQ-induced lung injury morphology

**Figure 1:**
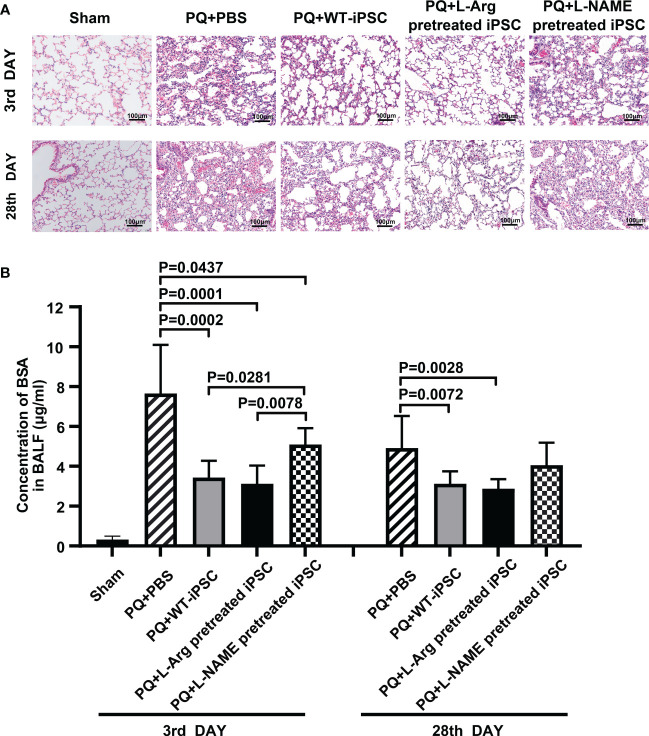
NO is necessary for iPSC to improve lung morphology and function. C57BL/6 mice received PQ or an equivalent volume of PBS by intratracheal instillation. The lungs were harvested 3 days and 28 days after intravenous iPSC injection. **(A)** Histologic changes were detected by hematoxylin and eosin staining; **(B)** fluorescein isothiocyanate (FITC)-labeled bovine serum albumin (BSA). The amount of FITC-BSA that diffused from the blood into the alveoli in the bronchoalveolar lavage fluid (BALF) represents the permeability of the pulmonary gas exchange membrane. The graph depicts the means ± standard deviation of the mean changes in five mice’s lungs.

We first verified the establishment of a PQ-induced ALI mouse model via hematoxylin and eosin staining. The data indicated that on the 3rd day, lungs exposed to PQ exhibited inflammatory cell infiltration and intra-alveolar hemorrhage. By the 28th day, the alveolar architecture was disrupted, interstitial tissue had thickened, and the respiratory membrane was almost destroyed. Both iPSCs and L-Arg pretreated iPSCs significantly ameliorated these parameters. whereas iPSC pretreated with L-NAME fails to alleviate lung injury. (**Figure 1A**).

### 3.2 NO is required for iPSCs to improve respiratory membrane dysfunction

The mice were challenged with PQ. After 3 d, respiratory membrane permeability was measured by analyzing the FITC-bovine serum albumin concentration in the BALF. PQ increased the FITC-BSA concentration by 61.98% at 3 d compared to the sham group. Compared with the PQ group, iPSC treatment decreased the BSA concentration by 55.09% and 36.54% on the third and 28^th^ day, respectively. Reducing the NO concentration in iPSCs led to the increase of BSA concentrations in L-NAME treated iPSCs. No differences were observed between iPSCs with or without pretreatment with L-arginine injected via the tail vein (**Figure 1B**).

### 3.3 NO promotes iPSCs to attenuate pulmonary fibrosis

**Figure 2:**
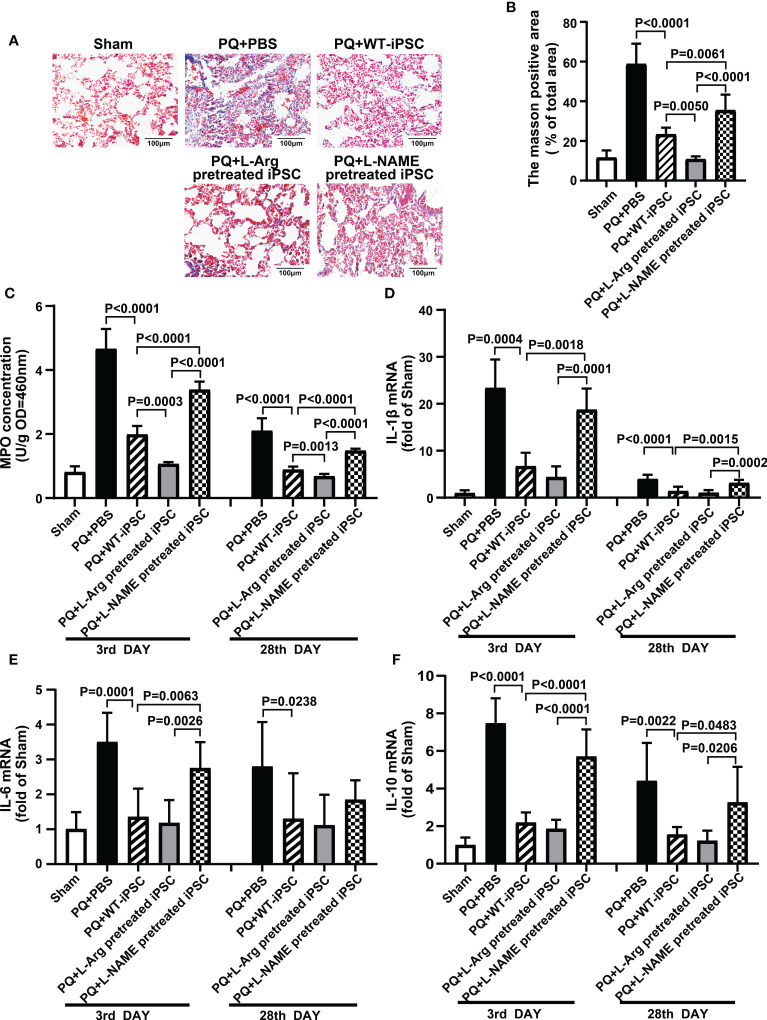
NO is required for iPSC attenuating PQ-induced lung inflammation in mice. **(A)** Representative photomicrographs of lung stained with Masson trichrome staining (black bar = 100 μm) and **(B)** percentage of collagen fiber content in lung tissue. **(C)** Inflammatory infiltration in lung tissue is shown based on myeloperoxidase (MPO) activity in lung tissue. The quantitative real-time polymerase chain reaction was performed for levels of mRNA encoding **(D)** IL-1β, **(E)** IL-6, and **(F)** IL-10 in lung tissues. Values are expressed as fold-change of gene expression compared to that sham group. Data are represented as means ± standard deviation; n = 5 mice/group.

PQ toxicity to the lung goes through an acute phase that lasts a few days, followed by a fibrosis phase that can persist for several weeks. To assess pulmonary fibrosis, Masson’s trichrome staining and the blue area fraction of the lung tissue were utilized (18, 19). iPSCs decreased fibrosis by 60.43% (blue area fraction) within 28 d compared to the PQ group. L-arginine-pretreated iPSCs significantly reduced the area of fibrosis by 81.64%. In contrast, L-NAME-pretreated iPSCs retained PQ-induced fibrosis, and the area of fibrosis was increased by 52.16% compared to the iPSCs group (**Figures 2A, B**).

### 3.4 NO is required for iPSCs to reduce myeloperoxidase activity and the expression of inflammatory factors in lung tissue

MPO is a marker of neutrophil infiltration in tissues. PQ increased MPO concentration from 0.81 ± 0.08 (Sham) to 4.66 ± 0.28 (PQ 3 d) and 2.10 ± 0.17 (PQ 28 d), which revealed neutrophil infiltration in the early phase of inflammation. On the third day, iPSCs decrease the MPO concentration by 57.87%; meanwhile, L-arginine-pretreated iPSCs decreased MPO concentration by 67.47% (**Figure 2C**).

The levels of IL-1β, IL-6, and IL-10 in the lung tissues of each experimental group are summarized in **Figures 2D–F**. Compared with the sham group, PQ treatment increased the expression of IL-1β, IL-6, and IL-10 23.37, 3.50, and 7.47 times, respectively, on the third day, while iPSC and L-arginine pretreatment significantly inhibited the levels of IL-1β, IL-6, and IL-10. Simultaneously, L-NAME-pretreated iPSCs failed to decrease PQ-induced IL-1β, IL-6, and IL-10 expression.

### 3.5 Intravenously delivered iPSCs trafficked to PQ-damaged lung

**Figure 3:**
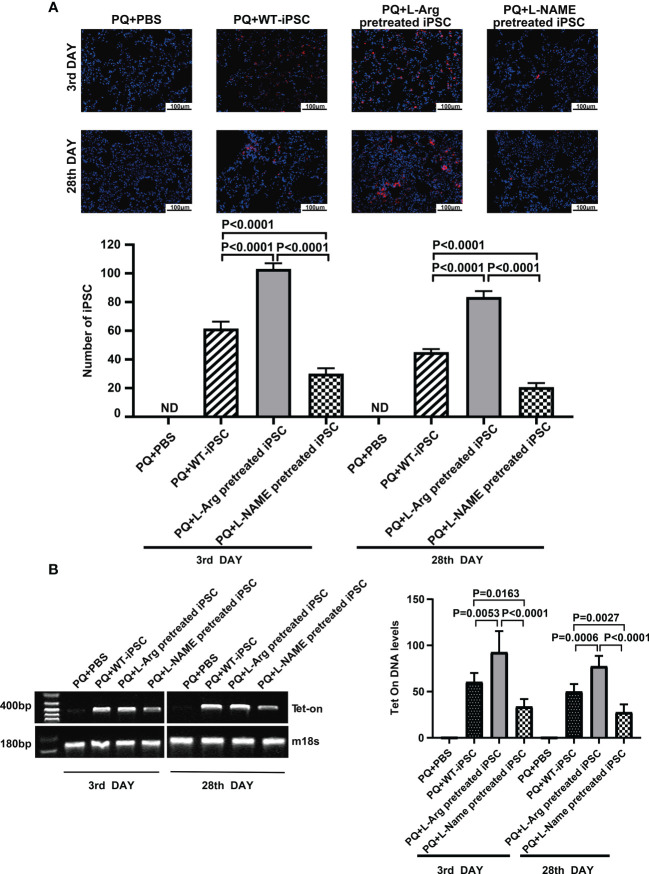
L-arginine promoted while L-NAME inhibited iPSC trafficking to the injured lung. **(A)** Representative fluorescence images of lungs harvested 3 or 28 days after paraquat injection along with PBS or iPSC administration. iPSCs were labeled with PKH26 (the red fluorescent dye) before injection. The slides were counterstained with DAPI. **(B)** RT-PCR analysis of Tet-on and m18s genes in the injured lung. Tet-on was detected in the lung of mice receiving iPSCs with or without L-arginine or L-NAME pretreatment but not from mice receiving PBS or undergoing sham operations. The relative amount of Tet-on DNA over m18s DNA in the injured lung. Data are represented as means ± standard deviation; n = 5 mice/group.

iPSCs were labeled with PKH26 (red fluorescent dye) before injection via the tail vein, and the dyed cells were observed in the injured lungs on the third and 28^th^ day through fluorescence microscopy. On the third day, the number of cells pretreated with L-arginine detected *in vivo* was increased from 61.40 ± 2.23 to 103.00 ± 1.79 compared to the non-treated group. On the day 28, the number of trafficked cells in the L-arginine-treated group increased from 45.00 ± 1.00 to 83.40 ± 1.89. Conversely, L-NAME blocked iPSCs *in vivo* by 51.14% and 54.22% compared to the iPSC group at days 3 and 28, respectively (**Figure 3A**).

At the same time, pulmonary tissue was harvested, and the number of the cells that homed to the injured lung tissue was detected via PCR of *Tet-on*, which was carried into the iPSCs using lentiviruses used for iPSC induction. Because *Tet-on* is expressed in iPSCs, but not in recipient cells, it can be used as a marker for iPSC quantification. The results confirmed that L-arginine significantly enhanced iPSC homing to the injured lung, and Tet-on expression increased by 53.41% and 54.45% on the third and 28^th^ day, respectively. In contrast, the L-NAME-pretreated group showed a decrease in *Tet-on* expression by 43.98% and 44.63%, respectively (**Figure 3B**).

### 3.6 NO affects the adhesion and migration ability of iPSCs

**Figure 4:**
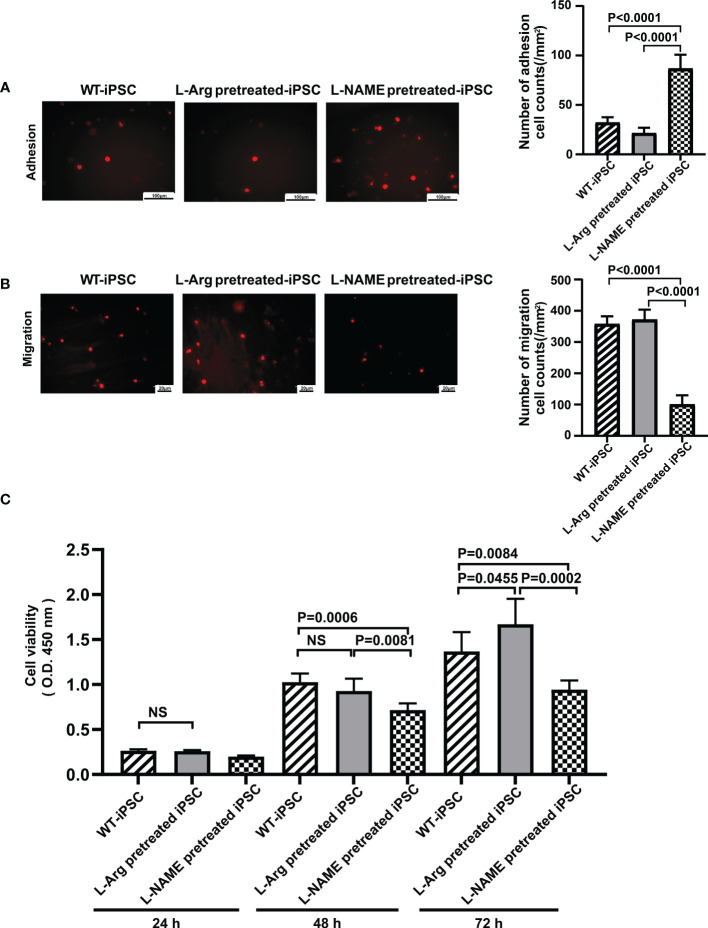
NO regulated iPSC viability, adhesion, and migration. L-NAME pretreatment promoted iPSC adhesion **(A)** and inhibited migration **(B)**. **(C)** Treatment (2 h) of iPSCs with L-NAME or L-arginine affects iPSC viability (n = 5). Data are represented as means ± standard deviation; n = 5 mice/group.

iPSCs were treated with 100 μM L-arginine or 100 μM L-NAME for 2 h, followed by labeling with PKH26. The adhesion ability of cells treated with L-arginine decreased by 33.33% (**Figure 4A**), while the migration only increased by 4.00% compared to untreated iPSCs (**Figure 4B**). Meanwhile, L-NAME-pretreated iPSCs promoted adhesion by 1.71 times and inhibited migration by 72.00%, which indicated that NO facilitates iPSC homing.

### 3.7 L-Arginine treatment promotes iPSC proliferation

iPSCs were pretreated with 100 μM L-arginine or 100 μM L-NAME for 2 h, and their proliferation ability was determined to reflect the influence of L-arginine or L-NAME on iPSC proliferation. As shown in **Figure 4C**, the cell proliferation rate in the L-arginine group increased by 13.96%, while L-NAME inhibited proliferation by 31.17% compared to untreated iPSCs at 72 h. No significant difference in the proliferation rate between untreated iPSCs and L-arginine-treated iPSCs was observed at 24 h and 48 h.

## 4 Discussion

Due to the high mortality and limited therapeutic options for lung injury, the development of new therapeutic strategies is critical. Thus, effective treatment strategies for lung injury will provide both short- and long-term benefits. iPSCs derived from the somatic cells of a patient were successfully introduced, with multidirectional differentiation potential similar to embryonic stem cells, a lack of ethical and moral controversies, and immunogenicity (20). Moreover, intravenously administered syngeneic iPSCs exert therapeutic effects on multiple diseases (21). iPSCs is regarded as an ideal cell-based therapies.

The pulmonary tissue is the primary target organ for PQ absorbed via the skin and digestive tract, due to the structure of PQ being similar to that of diamine and polyamine of the lung (22). Consequently, PQ competitively accumulates in the lung (23). The lung pathologic morphology of PQ toxicity is acute inflammatory infiltration and the development of lung fibrosis (24, 25). The current study demonstrated that the pathological manifestations of PQ-induced lung injury in mice involve damage to alveolar epithelial cells, widening of the lung septum, hemorrhage, inflammatory cell infiltration in the lung in the early stage, and evident fiber deposition in the late stage. Thus, a PQ-induced lung injury is the ideal model to evaluate the effect of iPSCs therapy.

iPSCs, administered intravenously following PQ intoxication, alleviated pathological morphology and partially decreased lung inflammation. The expression of *Tet-on* in iPSCs demonstrated that the transplanted iPSCs were recruited to the damaged pulmonary, and not detected in control mice. The number of iPSCs in the injured lung was associated with improved lung histological pathology and reduced cytokines. These data demonstrate that iPSCs have a therapeutic capacity for lung injury, although they did not restore damaged lung tissue, nor did they block the formation of pulmonary fibrosis. This is associated with the poor survival ability of stem cells within damaged tissues. Our previous research demonstrated that the persistent stimulation of iPSCs with H_2_O_2_ inhibits the trans-endothelial migration and adhesion of the cells, induces apoptosis, and limits their efficacy against lung injury (26). These findings indicate that pre-enhancing the biological function of iPSCs may improve their restorative action in damaged tissue and prolong the efficacy of iPSC-based treatment.

NO is a colorless endogenous gas with numerous functions that regulate physiological and pathophysiological effects. While NO is a crucial regulator of arterial conductance, platelet activation, nervous system function (27). excessive levels of this gas exacerbate the inflammatory response and tissue damage. While the NO inhibitor can reduce oxidative stress and improve cognitive deficits (28), the deletion of NO production exacerbates amyloid deposition and neuron loss in the hippocampus (29). Emerging evidence suggests that NO affects stem cell paracrine secretion, survival, migration, and immunosuppression (30, 31). In this study, L-arginine was used as the biological precursor of NO, and L-NAME, an inhibitor of nitric oxide synthase activity. Temporary treatment with L-arginine, lasting only two hours, did not affect the biological properties of iPSCs. Instead, NO-inhibitor pretreatment increased iPSC adhesion and inhibited migration, thereby obstructing the homing ability of iPSCs at the injury site. Interestingly, extracellular L-arginine enhanced iPSC proliferation at 72 h, whereas the NO inhibitor reduced the proliferation of iPSCs from as early as 48 h. These results demonstrated that NO is essential for maintaining the physiological effects of iPSCs, and that L-arginine supplementation maintains iPSC viability and improves cell survival. Consequently, we hypothesize that NO improves the restorative function of iPSCs and enhances the efficacy of iPSC therapy.

In the present study, we intravenously transplanted iPSCs pre-treated with/without NO precursor to injured lungs, induced by PQ. The results demonstrated that L-arginine dramatically enhances the migration of iPSCs to the damaged lung, as well as their survival, thus rendering an increased therapeutic effect that improves lung histology, and reduces pathological lung damage, alveolar epithelial capillary permeability, PMN influx into the alveolar lumen, and inflammatory cytokine levels, when compared with untreated iPSCs. The development of pulmonary fibrosis is mostly the result of acute lung injury (32). In this study, we demonstrated that L-arginine pretreated iPSCs could preserve lung architecture and prevent/reduce the occurrence of pulmonary fibrosis by effectively blocking early inflammation.

In conclusion, NO is essential for maintaining the biological behavior and function of iPSCs, and elevated NO levels in iPSCs improve the stem cell therapeutic efficacy on lung injury. Pretreating stem cells with L-arginine *in vitro* may be a reasonable strategy to enhance their long-term effects, and further study is needed to extend the observation time to 6 months or even longer and determine their potential for stopping or reversing pulmonary fibrosis.

## Data availability statement

The raw data supporting the conclusions of this article will be made available by the authors, without undue reservation.

## Ethics statement

The animal study was reviewed and approved by the animal care committee of Fudan University Shanghai Medical College.

## Author contributions

Conceptualization, MX and SC; Methodology, AC, SL, YL, DY, JH, XW, and NS; Writing-Original Draft, MX; Writing-Review & Editing, MX and SC; Funding acquisition, MX, SC, DY, NS and AC. All authors contributed to the article and approved the submitted version.
